# Drug-induced liver injury from antituberculous treatment: a retrospective study from a large TB centre in the UK

**DOI:** 10.1186/s12879-017-2330-z

**Published:** 2017-03-24

**Authors:** Aula Abbara, Sarah Chitty, Jennifer K. Roe, Rohma Ghani, Simon M. Collin, Andrew Ritchie, Onn Min Kon, John Dzvova, Harriet Davidson, Thomas E. Edwards, Charlotte Hateley, Matthew Routledge, Jim Buckley, Robert N. Davidson, Laurence John

**Affiliations:** 1 Department of Infection, Northwick Park Hospital, London North West Healthcare NHS Trust, London, UK; 2 Department of Immunology, University College London, London, UK; 3 School of Social and Community Medicine, University of Bristol, Bristol, UK; 4 Department of Chest and Allergy, St Mary’s Hospital, Imperial College Healthcare NHS Trust, London, UK; 5 NHLI, Imperial College London, London, UK

**Keywords:** Tuberculosis, Drug induced liver injury, Hepatotoxicity, Re-introduction regimen, Risk factors, Liver failure

## Abstract

**Background:**

We describe drug-induced liver injury (DILI) secondary to antituberculous treatment (ATT) in a large tuberculosis (TB) centre in London; we identify the proportion who had risk factors for DILI and the timing and outcome of DILI.

**Methods:**

We identified consecutive patients who developed DILI whilst on treatment for active TB; patients with active TB without DILI were selected as controls. Comprehensive demographic and clinical data, management and outcome were recorded.

**Results:**

There were 105 (6.9%) cases of ATT-associated DILI amongst 1529 patients diagnosed with active TB between April 2010 and May 2014. Risk factors for DILI were: low patient weight, HIV-1 co-infection, higher baseline ALP, and alcohol intake. Only 25.7% of patients had British or American Thoracic Society defined criteria for liver test (LT) monitoring. Half (53%) of the cases occurred within 2 weeks of starting ATT and 87.6% occurred within 8 weeks. Five (4.8%) of seven deaths were attributable to DILI.

**Conclusions:**

Only a quarter of patients who developed DILI had British or American Thoracic Society defined criteria for pre-emptive LT monitoring, suggesting that all patients on ATT should be considered for universal liver monitoring particularly during the first 8 weeks of treatment.

**Electronic supplementary material:**

The online version of this article (doi:10.1186/s12879-017-2330-z) contains supplementary material, which is available to authorized users.

## Background

Drug-induced liver injury (DILI) secondary to antituberculous treatment (ATT) is reported in 2–28% of patients [1, 2] varying with the definition, study population and treatment regimen. Risk factors associated with this potentially fatal complication include co-infection with HIV, hepatitis B or C, pre-existing chronic liver disease, high alcohol intake, malnutrition, advanced age, female sex and slow acetylators [1–11]. There is no agreed definition of TB DILI and most definitions focus on ALT. The DILI Expert Working Group have called for a consensus on the threshold criteria for any drug-related DILI, including ALT- and ALP-based criteria [12]. In cases of suspected DILI it is important to assess causality, for example, using scoring systems such as the Roussel Uclaf Causality Assessment Method (RUCAM) score [[9].

ALP-based criteria may not be appropriate in TB, given the granulomatous hepatitis picture associated with the infection itself [13]. Transaminitis with ATT may represent hepatic adaptation and occurs in up to 20% of patients receiving isoniazid monotherapy for latent TB [14–16]. A balance must therefore be struck between unnecessary cessation of ATT and responding to true DILI, given that the mortality of TB DILI can be up to 27% [17, 18].

Different recommendations have been made for monitoring liver tests (LTs) in patients on ATT by the British Thoracic Society [19] the American Thoracic Society (ATS) [14] and the European Respiratory Society (ERS) [20] with some favouring a risk factor based approach. Singanayagam et al. compared the two approaches, finding in favour of universal testing 2 weeks into treatment [2].

The DILI Expert Working Group [12] and DILIGEN study [21] use criteria based on ALT, ALP and bilirubin to guide cessation of ATT. All expert recommendations include treatment cessation at ALT > 5xULN or if the patient is icteric; ATS recommends cessation if ALT is 3-5xULN and the patient reports symptoms including nausea, anorexia, vomiting, abdominal pain and jaundice. Each advisory body has recommendations for treatment re-introduction when LTs have normalised; these either advise sequential re-introduction with incremental dose increase or re-introduction at full dosage [14, 19, 20].

Few UK studies have described LT monitoring for DILI, criteria for diagnosis and DILI outcomes on ATT [2, 22]. We sought to determine the sensitivity of symptom screen and risk factors in identifying DILI. In addition, we sought to describe the biochemical and clinical characteristics, clinical management and treatment outcomes for patients who develop abnormal LTs on ATT at one of the largest TB treatment centres in the UK.

## Methods

### Study design

We conducted a retrospective study of consecutive patients who developed DILI whilst on treatment for active TB at Northwick Park Hospital between April 2010 and May 2014. Patients with active TB were identified from the London TB register (LTBR) and cases with DILI were identified by case note review. For each patient with DILI, two cases of active TB without DILI who were treated during the same period (2011 to 2014) were selected at random from the LTBR. These were patients who had no abnormal LTs whilst on treatment. Patient data were extracted from hospital electronic databases and case records, including: demographic data, comorbidities, medications, symptoms, details of ATT, time of DILI onset from start of ATT, and timing and nature of reintroduction regimen. Risk factors investigated included age, sex, baseline LTs, HIV, hepatitis B and C, alcohol intake, chronic liver disease and drug dosage per kg body mass. Ethics approval was not required as data were collected routinely for clinical purposes.

### Study population

This study was carried out in the TB centre of Northwick Park Hospital, which is part of London North West Healthcare (LNWH) NHS Trust. The Trust saw an average of 600 TB patients annually from 2010 to 2014. It is located in an ethnically diverse, urban area with a low-to-medium prevalence of HIV, hepatitis B and C infection.

### Patient inclusion criteria and DILI definitions

We designated patients with a positive TB culture as ‘confirmed cases’ and those with negative culture with clinical, histological and radiological features of TB and who improved on treatment as ‘presumed cases.’

We identified and categorised possible DILI (pDILI) cases any time after the start of ATT based on DILI expert working group [6] and DILIGEN criteria [15]. These were as follows: type 1 - pDILI ALT >3xULN, type 2 - pDILI ALP >2xULN and bilirubin >1xULN, and type 3 pDILI an isolated bilirubin >2xULN. We subdivided type 1 cases into patients with ALT >5xULN without symptoms (type 1a) and those with ALT 3-5xULN with symptoms (type 1b). In a risk factor analysis, DILI was defined according to the BTS and ATS DILI guidelines using the type 1a and type 1b definitions which are ALT based; this was chosen due to ALP based criteria potentially being affected by TB infection related cholestasis and hence may not be indicative of true DILI [13]. At the time of sampling, the ULNs in our laboratory were ALT 55 IU/L, ALP 150 IU/L and bilirubin 21 umol/L. No patients were excluded from the analysis.

### Causality assessment

Other causes of liver injury were excluded with a viral and autoimmune screen, an ultrasound of the liver and the RUCAM score was calculated for each patient [9].

### Monitoring and management of LTs during study period

Standard of care for the duration of the study was physician and TB nurse review at diagnosis with education about ATT side-effects. Baseline LTs were performed and all patients were offered HIV, hepatitis B and C screening. The next physician review at two to 4 weeks and at each subsequent visit included questioning about symptoms of DILI: nausea, vomiting, abdominal pain, dark urine, itching and loss of appetite. There was no routine monitoring of LTs unless symptoms were reported.

Patients with abnormal LTs were managed according to local guidelines, which are based on BTS [19] and ATS [14, 23] guidance. If the ALT was 3-5xULN with symptoms or >5xULN without symptoms, ATT was stopped or altered. Those with asymptomatic ALT 3-5xULN either had their ATT altered or were monitored closely depending on physician preference. In the event of severe TB, we gave a non-hepatotoxic background regimen (usually ethambutol plus amikacin) whilst stopping rifampicin, isoniazid and pyrazinamide. LTs were checked at intervals until ALT was normal or approaching normal. Treatment was re-introduced using a staggered incremental dose increase regimen with omission of pyrazinamide and consequential extension of treatment to 9 months.

#### Details of re-introduction regimen

Re-introduction was as follows: day 1 onwards, normal dose ethambutol plus: days 1–3 isoniazid 50 mg once daily (OD); days 4 onwards, isoniazid 300 mg OD; plus: days 7–9 rifampicin 75 mg OD; days 10–12 rifampicin 300 mg OD; day 13 onwards, normal doses of rifampicin, isoniazid and ethambutol. LTs were monitored during the re-introduction at days 0, 3, 6, 9, 12 and 15. We did not re-introduce pyrazinamide, and treatment was extended to 9 months for that reason.

### Statistical analysis

Baseline characteristics of cases and controls were compared using Fisher’s exact and Kruskal-Wallis tests. Logistic regression was used to estimate crude odds ratios for each potential risk factor. Odds ratios were estimated adjusted for age, sex and baseline levels of ALT, ALP and bilirubin as a priori confounders. We investigated whether peak ALT was associated with time from stopping to reintroduction and with time from reintroduction to being established on full dose treatment, using linear regression models adjusted for age, sex and baseline ALT. All analyses were performed using Stata 13 (Stata Statistical Software: Release 13. StataCorp).

## Results

**Fig. 1:**
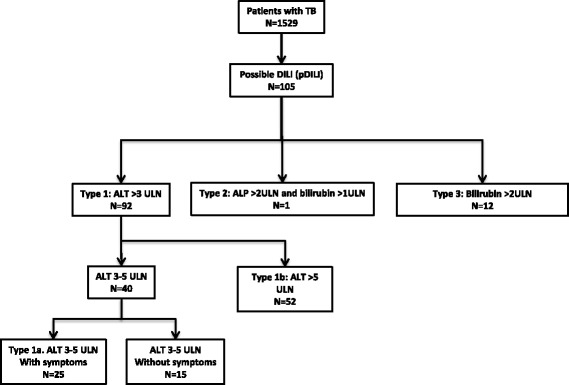
A flow chart showing the number of patients in the original cohort, those with possible DILI (pDILI) and those with DILI according to our local guidelines which are based on BTS and ATS criteria; DILI 1a and DILI 1b were used for the risk factor analysis. ULN = Upper Limit of Normal. ULN for ALT was 55 IU/L, for ALP 150 IU/L and for bilirubin 21umol/L

We identified 105 (6.9%) cases of pDILI among 1529 patients diagnosed with confirmed or presumed TB between April 2010 and May 2014 (Fig. 1). Among the pDILI cases, 92 (87.6%) had type 1 pDILI, one (0.1%) had type 2 pDILI, and 12 (11.4%) had type 3 pDILI. Seventy-seven of the pDILI cases fulfilled the ATS/BTS criteria for TB DILI: 52 (49.5%) type 1a and 25 (23.8%) had type 1b DILI.

### RUCAM analysis

The RUCAM scores for causation of ATT are as follows: all patients included scored 3 or above (possible relationship to DILI). 45 (42.9%) had a score of 3–5 (possible,) 58 (55.2%) had a score of 6–8 (probable) and 2 (1.9%) had a score of ≥9 highly probable. The median (IQR) of RUCAM scale was 4 (4–6).

The characteristics of the pDILI cases are summarised in Table 1. Initial treatment regimens are detailed in Additional file 1: Table S1.

**Table 1:** Baseline characteristics of all cases of possible DILI (pDILI)

Characteristic		n (%)
pDILI		105
Sex	Female	49 (46.7)
	Male	56 (53.3)
Age (years)	18–24	17 (16.2)
	25–29	22 (21.0)
	30–34	10 (9.5)
	35–39	13 (12.4)
	40–49	14 (13.3)
	50–59	12 (11.4)
	60+	17 (16.2)
Ethnic origin	Indian	55 (52.4)
	Pakistani	8 (7.6)
	Nepalese	8 (7.6)
	Afghan	2 (1.9)
	Somali	4 (3.8)
	Romanian	2 (1.9)
	Black	6 (5.7)
	White	11 (10.5)
	Asian (Other)	7 (6.7)
	Other	2 (1.9)
Site of tuberculosis		
Pulmonary		21 (20)
Extrapulmonary		61 (58.1)
Both		23 (21.9)
Total culture confirmed		62 (59)
Fully sensitive TB		56 (53.3)
Isoniazid monoresistant TB		3 (2.9)
Multi-drug resistant TB		3 (2.9)
HIV positive		8/96 (7.6)
HCV positive (detectable RNA)		3/98 (3.0)
HBV positive		0/101 (0)
Alcohol Excess^a^		9/105 (8.6)
Chronic Liver Disease^b^		7/105 (6.7)
No risk factor^c^		78/105 (74.2)
Other risk factors		Median (IQR), n
Baseline ALT (IU/L) (normal range 10–50)		24 (17–32), *n* = 77
Baseline ALP (IU/L) (normal range 30–130)		98 (75–135), *n* = 77
Baseline BILI (umol/L) (normal range 0–21)		8 (6–12), *n* = 77
Weight (kg)		53.9 (48.0–65.0), *n* = 74
Rifampicin dose per kg	(if given)	9.98 (9.00–11.1), *n* = 74
Isoniazid dose per kg	(if given)	5.57 (4.62–6.25), *n* = 74
Pyrazinamide dose per kg	(if given)	25.8 (22.7–29.8), *n* = 74
Moxifloxacin dose per kg	(if given)	7.34 (6.15–8.15), *n* = 36

^a^Alcohol excess is defined as >21 units per week for men or >14 units per week for women. ^b^Causes of chronic liver disease are as follows: hepatitis C (3), alcoholic liver disease (2), autoimmune hepatitis (1) and 1 drug related, secondary to methotrexate. ^c^As defined by ATS/BTS

### Symptoms

Seventy-one (67.6%) patients had the nature of their symptoms recorded at the time of pDILI, the commonest being nausea and vomiting in 57 (54.3%), abdominal pain in 19 (18.1%), skin complaints in 18 (17.1%) and clinical jaundice in 13 (12.4%) (Additional file 1: Table S1).

### Management and outcomes of pDILI

**Fig. 2:**
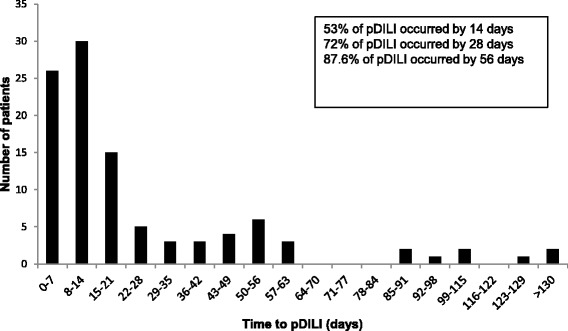
This bar chart shows the time to pDILI from the time of starting anti-TB treatment

Median time to onset of pDILI was 12.5 (IQR: 7–30) days (Fig. 2). Sixty-three (60%) patients stopped ATT; 11 of these received ethambutol and amikacin before re-introduction. 11 (10.5%) stopped pyrazinamide only, of whom five were given a quinolone (moxifloxacin or levofloxacin) as a replacement for pyrazinamide, 27 (25.7%) patients did not stop treatment and four (3.8%) had other interventions.

Full details of the management of the 63 patients who stopped treatment are provided in Additional file 1: Table S1.

The median time from stopping treatment to being re-established on full-dose treatment was 28 days (IQR 24–35): median time from stopping to starting re-introduction was 18 days (IQR 14–28); from re-introduction to being established on full-dose treatment 14 days (IQR 9–22). Time from stopping to re-introduction and time from re-introduction to being established on full-dose treatment increased as peak ALT increased: a mean increase of 7.1 (95% CI 1.0, 13.2) days (*p* = 0.02) per unit increase in log10 peak ALT and 7.1 (95% CI 2.0, 12.3) days (*p* = 0.01) per unit increase in log10 peak ALT (adjusted for age, sex and baseline ALT).

Of the 27/105 (25.7%) patients who did not stop treatment, seven had type 3 pDILI (bilirubin 41-54umol/L) and ten had ALT 3-5xULN but no symptoms hence did not meet the criteria for stopping treatment. These patients settled without intervention. Five patients had ALT >5xULN and five had ALT 3-5xULN with symptoms. Of these ten, three had abnormal liver tests at baseline which worsened then improved on treatment, six were monitored more carefully and one met the criteria for type 2 pDILI and settled with monitoring. In all ten patients, pDILI settled; eight completed treatment and two were lost to follow-up.

Of the 105 pDILI cases, 59 (56.2%) were successfully re-established on treatment; 25 (23.8%) resolved without stopping treatment, 13 (12.4%) had a second pDILI, three (2.9%) were lost to follow up at time of DILI and five (4.8%) died with liver injury as a probable contributing cause of death.

With regards to outcome of TB, 79 (75.2%) completed treatment and were cured, seven (6.7%) died, two (1.9%) discontinued treatment, ten (9.5%) transferred to another centre and seven (6.7%) were lost to follow up (Additional file 1: Table S1).

Of the 13 patients who had more than one episode of pDILI, two had a history of alcohol excess, one had hepatitis C and ten had no identified risk factors. Eight of these recurrent pDILI cases completed TB treatment, two died, one was transferred out to another centre and the remaining two did not complete treatment as intended.

### Hospitalisations and deaths

There were 34 hospital admissions amongst 32 patients (30% of total) with a median inpatient stay of 14 days (IQR 7–21).

Of the seven (6.7%) deaths, the mean age was 55.1 years at diagnosis and mean baseline weight was 65.1 kg. One patient had HIV-1 co-infection, one had myeloma, one had chronic renal failure, one had severe emphysema, one was diabetic and had previous breast cancer. Five of the seven were of South Asian origin. None had a history of alcohol excess, chronic liver disease, hepatitis B or C, or were on other hepatotoxic drugs. Three had pDILI within 2 weeks and one had recurrent pDILI; multiple attempts were made to restart their treatment given the disseminated nature of their TB. Two had pulmonary TB, three had extrapulmonary TB and two had both. Five received standard quadruple therapy, one had ethambutol substituted with moxifloxacin due to renal failure and one received an undocumented regimen. Two of the seven fatal cases reported jaundice and had skin complaints at the time of their pDILI and five reported no symptoms. One patient died of liver failure whilst awaiting liver transplant; they were 42 years old and HIV-1 co-infected. The patient developed a widespread rash and a significant transaminitis with a peak ALT of 1692 IU/L, a peak ALP of 509 IU/L and a peak bilirubin of 148 umol/L (0–21). There was also an eosinophilia and was diagnosed with DRESS syndrome. The other patients were considered but not referred for transplantation either because they did not fulfil the criteria for liver transplantation or had absolute or relative contraindications or significant comorbidities that precluded transplantation; they received supportive clinical care. Five of the seven died with pDILI being a likely contributor to death giving an attributable mortality of 4.8%.

### DILI risk factors

As previously described, 77 patients had ALT 3-5xULN with symptoms or an ALT >5xULN, thereby meeting ATS/BTS criteria for stopping TB therapy. There were no baseline differences in sex, age or ethnicity between DILI cases and controls. Baseline ALP was positively associated with a risk of DILI with an adjusted odds ratio (AOR) 7.33 (95% CI 1.46–36.8) per 10-fold increase in ALP, (*p* = 0.03) though median values for both groups remained within the normal range (Table 2). Patient weight was inversely associated with reduced risk of DILI (AOR 0.96 (0.94–0.99) per kg, *p* = 0.003). Alcohol intake (any amount) was associated with an increased risk of DILI (AOR 5.94 (95% CI 2.34–15.1), *p* < 0.001). Higher drug doses (per kg body weight) were associated with an increased risk of DILI: isoniazid, AOR 1.77 (1.30–2.40) per 1 mg/kg increase in dose (*p* < 0.001); pyrazinamide AOR 1.04 (1.00–1.09) per 1 mg/kg increase in dose (*p* = 0.04). This dose-response effect was not seen for rifampicin or moxifloxacin. The small number (eight) of HIV-1 co-infected patients had a four-fold higher risk of DILI (AOR 4.40 (1.06–18.3), p 0.04) (Tables 2 and 3). No positive association was found between increasing age and risk of DILI.

**Table 2:** Characteristics of pDILI Type 1 cases (BTS/ATS criteria) and controls

Characteristic		Controls	DILI cases	
		Median (IQR)	Median (IQR)	*P*-value^†^
Baseline ALT (IU/L)		19 (14–29), *n* = 185	24 (17–32), *n* = 77	0.03
Baseline ALP (IU/L)		86 (71–102), *n* = 187	98 (75–135), *n* = 77	0.03
Baseline bilirubin (umol/L)		8 (5–10), *n* = 185	8 (6–12), *n* = 77	0.28
Weight (kg)		61.2 (54.5–69.6), *n* = 178	53.9 (48.0–65.0), *n* = 74	0.001
Rifampicin dose per kg	(if given)	9.68 (8.62–10.7), *n* = 178	9.98 (9.00–11.1), *n* = 74	0.17
Isoniazid dose per kg	(if given)	4.85 (4.31–5.45), *n* = 178	5.57 (4.62–6.25), *n* = 74	<0.001
Pyrazinamide dose per kg	(if given)	24.4 (21.4–27.5), *n* = 178	25.8 (22.7–29.8), *n* = 74	0.05
Moxifloxacin dose per kg	(if given)	6.15 (5.71–7.04), *n* = 21	7.67 (5.71–8.00), *n* = 10	0.20
		% (fraction)	% (fraction)	
HIV positive		2.2 (4/185)	7.3 (5/69)	0.06
HCV positive		1.7 (3/179)	1.4 (1/73)	1.00
HBV positive		2.2 (4/180)	0.0 (0/74)	0.33
Alcohol consumption^a^	Nil	93.3 (167/179)	71.4 (40/56)	<0.001
	Any	6.7 (12/179)	28.6 (16/56)	
Chronic Liver Disease		2.2 (4/186)	3.9 (3/77)	0.42

^†^ *P*-values from Kruskal-Wallis test (medians), Fisher’s exact test (proportions)

^a^ Most patients drank no alcohol and only 1 patient (male) drank in excess of 21 units/week; given the small number of high alcohol intake, this was analysed as no intake versus any intake of alcohol

**Table 3:** Odds ratios for exposures among pDILI Type 1 cases compared with controls

		Crude odds ratio (95% CI)	Adjusted odds ratio (95% CI)^a^
Log baseline ALT (IU/L)		2.28 (0.96, 5.42)	1.96 (0.76, 5.04)
Log baseline ALP (IU/L)		6.67 (1.50, 29.7)	7.33 (1.46, 36.8)
Log baseline BILI (umol/L)		1.93 (0.56, 6.67)	2.20 (0.60, 8.01)
Weight (kg)		0.97 (0.95, 0.99)	0.96 (0.94, 0.99)
Rifampicin dose per kg		1.10 (0.93, 1.31)	1.12 (0.93, 1.35)
Isoniazid dose per kg		1.62 (1.22, 2.13)	1.77 (1.30, 2.40)
Pyrazinamide dose per kg		1.04 (1.00, 1.08)	1.04 (1.00, 1.09)
Moxifloxacin dose per kg		1.52 (0.83, 2.78)	1.53 (0.72, 3.27)
HIV		3.50 (0.91, 13.4)	4.40 (1.06, 18.3)
Alcohol	Nil	1.00 (reference)	1.00 (reference)
	Any	6.00 (2.59, 13.9)	5.94 (2.34, 15.1)

^a^ Adjusted for age, sex and baseline ALT, ALP and bilirubin

## Discussion

Only 25.7% of patients who developed pDILI met BTS/ATS criteria at baseline. At 6.9%, the rate of ATT-associated pDILI in this cohort falls at the lower end of the estimates across different studies with rates reported between 2% and 28% [1, 2, 8, 24–26], and is very similar to a recent study from the UK that reports a rate of 7.3% [2]. More than half (53%) of pDILI occurred in the first 2 weeks and 87% occurred within the first 2 months of starting ATT. The duration of interruption of ATT in patients with pDILI was related to peak ALT and therefore may be shortened by earlier identification of abnormal LTs through universal rather than risk factor based monitoring.

The definition of ATT-associated DILI at this centre follows BTS and ATS guidance. The DILI expert working group [12] called for standardisation of DILI phenotypes, including types based on ALP and bilirubin criteria. This may not be appropriate for TB given that it can cause a granulomatous hepatitis with raised ALP [13], and a rising ALP on treatment may indicate a paradoxical reaction rather than true DILI [12]. Of our six cases with ALP >2xULN, three had an elevated ALP at baseline; the ALP increased in all during treatment and all except one had an associated ALT rise. Though it may not be a useful marker in isolation, higher ALP at baseline was an independent risk factor for DILI in our study however it remained well within the normal range.

Isolated hyperbilirubinaemia does not fulfil the DILI working group definition of DILI though BTS guidance suggests monitoring and potentially stopping treatment [19]. Some investigators report that hyperbilirubinaemia is an independent predictor of mortality in patients with DILI [17]. This is due to the fact that patients with significant hepatocyte dysfunction may not produce an ALT rise and hyperbilirubinaemia may be a late manifestation. In our cohort, seven of 12 patients who fulfilled this definition had a bilirubin only slightly above twice the ULN of bilirubin with a range between 41 and 54 umol/L. This was self-limiting and may reflect the effect of rifampicin.

In guidelines defining DILI, the ULN of ALT is often not defined and there is high inter-laboratory variability [14, 19, 27]. This variability can significantly alter estimates of DILI and hamper understanding and management of TB DILI [3, 27] as well as confounding comparisons between centres. Values of ALT based on weight and gender may be more useful.

Attribution of DILI to a particular medication can be difficult; this is where scoring systems including RUCAM are useful. In our study, all included patients were scored as at least ‘possible’ and more than half of the patients scored ‘probable’ relationship to DILI secondary to ATT. The RUCAM score takes into account the time to onset of injury after starting the potentially causative drug, course of DILI after stopping the drug, specific risk factors, other medications which can cause DILI, exclusion of other causes of liver disease, known potential for DILI of the drug in question and response to rechallenge [9, 10, 28]. In ATT related DILI the lack of response to rechallenge may not be against the causative role of ATT as rechallenge can be successful [1, 12] and is advised by some experts [9, 14, 19].

The commonest symptoms our DILI patients reported were nausea and vomiting, affecting 54.3%. Gastric intolerance frequently occurs with ATT, especially pyrazinamide, however we advise a low threshold for LTs in patients with these complaints. Jaundice was lower (12.4%) among our patients than in other centres [17, 29, 30] who reported a higher mortality possibly reflecting earlier diagnosis in our cohort.

There is no clear evidence as to whether staggered re-introduction with incremental dose increase, or restarting drugs at full dose, is preferable [14, 19, 31–34]. Incremental dose increase results in longer without full treatment, but may be safer and allow for hepatic adaptation. Among our pDILI patients, the median time from stopping treatment to re-establishment on full dose ATT was 28 days (IQR 24–35), a significant interruption in treatment. The duration of interruption was related to the peak ALT and therefore may be shortened by earlier identification of DILI. 75.2% of our patients successfully completed a regimen containing at least RHE; others report higher completion rates [35] though differences in DILI definition [1, 12, 14, 19] and patients lost to follow up or transferred to other centres mean our completion rate may be higher.

We found that HIV co-infection, lower weight, alcohol intake and higher baseline ALP were associated with increased risk of DILI; other risk factors were not associated with DILI. Most notably, increasing age was not found to be a risk factor which is consistent with other studies [5, 8]. This may be because only 16.2% of cases were over 65 years of age and because older patients are considered at high risk for DILI, we often omitted pyrazinamide in these patients and monitored them more carefully for side effects [14]. Alcohol excess has been reported as a risk factor in other studies however in our study, we report alcohol intake as a risk factor given the low occurrence of alcohol excess in our cohort [1, 14, 36]. Given that 74.3% of patients did not meet ATS/BTS criteria, we agree with Singanayagam et al. that targeted monitoring of DILI in those with these risk factors is an insensitive strategy and can result in delayed diagnosis [2].

In our study, 53% of pDILI occurred within 2 weeks and 87% by 2 months. The median time to pDILI of 12.5 days (CI 7–30) is comparable with other reports [2, 24, 25] and indicates to us that all patients should have LTs 2 weeks after starting ATT, even if asymptomatic. Given the frequency of ATT-associated DILI, regular monitoring of LTs in the first 2 months of treatment would capture the vast majority of cases as is recommended for other hepatotoxic drugs such as DMARDs (disease modifying anti-rheumatic drugs.) [37].

Recently published 2016 NICE guidelines do not have clear guidance on the monitoring of LTs to detect hepatotoxicity in patients with active TB [38], although they do have recommendations for the sequential, full-dose re-introduction of ATT in those who have a treatment interruption due to hepatotoxicity.

Limitations of this study include its retrospective nature, meaning that monitoring for occurrence of pDILI could have varied over time, though this is unlikely given that the TB specialists managing patients remained the same during this period. It would also have contributed to incomplete recording of symptoms. Rates of pDILI could be higher given the possibility of asymptomatic pDILI; those patients could be identified if there was universal LT testing of all patients regardless of symptoms. Although our definitions of ATT-associated DILI followed BTS and ATS guidance, it is possible that some DILI cases may not be attributable to ATT. Ideally, a replication study would have a multi-centre, prospective design and use the RUCAM scale as part of the assessment of causality. Our study describes data from a single centre, and would benefit from replication. However, this is a large study, is representative of the UK TB cohort and is one of the few recent UK-based studies that provides relevant data which characterises DILI in the UK.

## Conclusions

In this study, we demonstrate an overall pDILI rate of 6.9% in this cohort and mortality with pDILI as a contributor in 4.8%. We have not found a risk factor screen approach useful with only 25.7% meeting this criteria. Though there are short comings, this study indicates that monitoring of LTs during the first 2 months of ATT should identify the majority of pDILI earlier, possibly shortening treatment interruption and reducing mortality.

## Additional file

Additional file 1: Table S1. Characteristics of pDILI. This table contains details of the 105 patients including the kinetics of pDILI, symptoms at presentation, initial regimen, management and overall outcome. (DOCX 20 kb)

## Abbreviations

AOR: Adjusted odds ratio
ATS: American thoracic society
ATT: Antituberculous therapy
BTS: British thoracic society
DILI: Drug-induced liver injury
DMARDS: Disease modifying anti-rheumatic drugs
ERS: European respiratory society
IQR: Inter-quartile range
LT: Liver test
LTBR: London tuberculosis register
RUCAM: Roussel Uclaf Causality Assessment Method
TB: Tuberculosis
ULN: Upper limit of normal
