# Supplementary material for: Drug-induced liver injury from antituberculous treatment: a retrospective study from a large TB centre in the UK
Source: BMC Infect Dis. 2017 Mar 24;17:231. doi: 10.1186/s12879-017-2330-z (PMC5366108; doi:10.1186/s12879-017-2330-z)
Supplement: Additional file 1: Table S1. — Characteristics of pDILI. This table contains details of the 105 patients including the kinetics of pDILI, symptoms at presentation, initial regimen, management and overall outcome. (DOCX 20 kb) [file 12879_2017_2330_MOESM1_ESM.docx]

Additional file 1: **Table S1** Characteristics of pDILI (N=105 unless otherwise stated)

|  |  | | | Median (IQR) |
| --- | --- | --- | --- | --- |
| **Timing of pDILI (n=104)** |  | | |  |
| Time to onset of pDILI (criteria 1^st^ met, days) | | | | 12.5 (7 – 30) |
| **In those who stopped treatment (n=63)** | | | |  |
| Time from stopping to starting re-introduction | | | | 18 (14-28) |
| Time from starting re-introduction to full establishment | | | | 14 (9-22) |
| Time from stopping treatment to full establishment | | | | 28 (24-35) |
| **Symptoms at presentation (n=105)** |  | | | n (%) |
| Total with symptoms recorded |  | | | 71 (67.6) |
| Nausea/Vomiting |  | | | 57 (54.3) |
| Abdominal pain  Skin |  | | | 19 (18.1)  18 (17.1) |
| Jaundice |  | | | 13 (12.4) |
| Fever |  | | | 7 (6.7) |
| Confusion |  | | | 3 (2.9) |
| **Initial regimen** |  | | |  |
| RHZE |  | | | 86 (81.0) |
| RHZM |  | | | 12 (11.4) |
| RHME |  | | | 3 (2.9) |
| RHZME |  | | | 1 (0.9) |
| RHZ |  | | | 1 (0.9) |
| RHE |  | | | 1 (0.9) |
| Unknown |  | | | 1 (0.9) |
| **Management** |  | | |  |
| Stop all |  | | | 63 (60.0) |
| Stop Z |  | | | 6 (5.7) |
| Stop Z, add quinolone |  | | | 5(4.8) |
| Not Stopped |  | | | 27(25.7) |
| Other |  | | | 4(3.8) |
| **Re-introduction regimen for those who stopped treatment completely** (n=63) | | | |  |
| RHE | |  | | 31 (49.2) |
| RHEM | |  | | 15 (23.8) |
| RHM | |  | | 3 (4.8) |
| REM | |  | | 3 (4.8) |
| RHEA | |  | | 1 (1.5) |
| RHZM | |  | | 1 (1.5) |
| RHEAM | |  | | 1 (1.5) |
| MDR Treatment | |  | | 1 (1.5) |
| Not restarted* | |  | | 7 (11.1) |
| **pDILI outcome** | |  | |  |
| Successfully re-established on treatment | |  | | 59 (56.2) |
| Resolved without stop | |  | | 25 (23.8) |
| 2^nd^ pDILI | |  | | 13 (12.4) |
| Lost to follow-up | |  | | 3(2.9) |
| Died (pDILI contributing) | |  | | 5 (4.7) |
| **Overall Outcome** | |  | |  |
| Completed | |  | | 79 (75.2) |
| Transferred to another centre | |  | | 10 (9.5) |
| Lost to follow-up | |  | | 7 (6.7) |
| Died | |  | | 7 (6.7) |
| Treatment discontinued | |  | | 2 (1.9) |
| **Admissions** | |  | |  |
| Number of admission, n (%) | |  | | 34 (32.4) |
| Median inpatient days, n (IQR) | |  | | 14 (7-21) |
| **Blood withdrawals from start to end of treatment** | | |  | n (%) |
| 0 | |  | | 2 (1.9) |
| 1 - 2 | |  | | 24 (22.9) |
| 3 - 6 | |  | | 26 (24.8) |
| 7+ | |  | | 15 (14.3) |
| N/A or unknown | |  | | 38 (36.2) |

*In 7 patients, ATT was not restarted: four of these were re-challenged with isoniazid but had a second episode of pDILI (two with rash), in one case it was decided that enough treatment had already been given, and two patients died before their LFTs settled.
